# Prehospital critical care beyond advanced life support for out-of-hospital cardiac arrest: A systematic review

**DOI:** 10.1016/j.resplu.2024.100803

**Published:** 2024-12-12

**Authors:** Adam J. Boulton, Rachel Edwards, Andrew Gadie, Daniel Clayton, Caroline Leech, Michael A. Smyth, Terry Brown, Joyce Yeung, on behalf of the International Liaison Committee on Resuscitation (ILCOR) Education, Implementation and Team (EIT) taskForce

**Affiliations:** a Warwick Clinical Trials Unit, Warwick Medical School, University of Warwick, Coventry, UK; b West Midlands CARE Team & Emergency Department, University Hospitals Birmingham NHS Foundation Trust, Birmingham, UK; c Critical Care Unit, University Hospitals Birmingham NHS Foundation Trust, Birmingham, UK; d Emergency Department, University Hospitals Coventry and Warwickshire NHS Trust, Coventry, UK; e Applied Research Collaboration West Midlands, Warwick Medical School, University of Warwick, Coventry, UK

**Keywords:** Prehospital physician, Critical care paramedic, Emergency Medical Services, Ambulances, Cardiac/heart arrest, Resuscitation

## Abstract

**Aim:**

To assess the clinical outcomes of patients with out-of-hospital cardiac arrest attended by prehospital critical care teams compared to non-critical care teams.

**Methods:**

This review was prospectively registered with PROSPERO and the eligibility criteria followed a PICOST framework for ILCOR systematic reviews. Prehospital critical care was defined as any provider with enhanced clinical competencies beyond standard advanced life support algorithms and dedicated dispatch to critically ill patients. MEDLINE, Embase and CINAHL databases were searched from inception to 20 April 2024. Risk of bias was assessed using the ROBINS-I tool and the certainty of evidence by the GRADE approach. Meta-analyses of pooled data from studies at moderate risk of bias were performed using a generic inverse-variance with random-effects.

**Results:**

The search returned 6,444 results and 17 articles were included, reporting 1,192,158 patients. Three studies reported traumatic patients and one reported paediatric patients. All studies were non-randomised and 15 were at moderate risk of bias. Most studies included prehospital physicians (n = 16). For adult non-traumatic patients, the certainty of evidence was low and prehospital critical care was associated with improved survival to hospital admission (OR 1.95, 95% CI 1.35–2.82), survival to hospital discharge (OR 1.34, 95% CI 1.10–1.63), survival at 30 days (OR 1.56, 95% CI 1.38–1.75), and favourable neurological outcome at 30 days (OR 1.48, 95% CI 1.19–1.84). Prehospital critical care was also associated with improved outcomes for traumatic and paediatric patients and the certainty of evidence was very low.

**Conclusion:**

Attendance of prehospital critical care teams to patients with out-of-hospital cardiac arrest is associated with improved outcomes.

## Highlights

• Prehospital critical care teams treat OHCA patients in international EMS systems.
• Nearly all published prehospital critical care teams include physicians.
• Over 1.1 million patients are reported, most from registries in Japan.
• Prehospital critical care is associated with improved outcomes in cardiac arrest.
• There is very low certainty of evidence for traumatic and paediatric arrests.

## Introduction

The emergency medical services (EMS) response is a critical element in the pathway of care for out-of-hospital cardiac arrest (OHCA) patients.^1^, ^2^ The optimal configuration of EMS systems is unclear and varies between countries.^3^ Many countries utilise prehospital critical care teams (CCT) as part of a tiered EMS response. These teams are specialists in care of the critically ill patient and have greater exposure to resuscitation, offering potential clinical benefits.^4^ These teams have competencies beyond that of standard EMS teams delivering advanced life support (ALS), including advanced airway management, prehospital emergency anaesthesia, sedation/paralysis, blood transfusion, central venous access, inotropes/vasopressors, invasive blood pressure monitoring, mechanical ventilation, surgical procedures, and diagnostic ultrasound.^5^ CCTs respond by helicopter or rapid response vehicle and can support transfer over extended distances, which may allow patients to receive hospital-level care at a more optimal facility. They often attend in addition to an ALS team or may attend in isolation dependent on the setting. Understanding the clinical efficacy associated with CCTs is important to inform the development of services and how these teams are utilised within the emergency care system.

A prior systematic review and meta-analysis searched the literature to June 2014 and compared physician-guided resuscitation with paramedic-guided resuscitation.^6^ The paramedic-guided control group included studies where basic life support was provided. ALS is recommended by guidelines and has become the standard of care, and hence does not represent current clinical practice in many international EMS systems.^7^, ^8^ Another systematic review did use ALS support as the control group and searched the literature to April 2016.^9^ Six studies were retrieved and meta-analysis was not performed. Since then a number of large studies have been published from multiple international EMS systems.^10^, ^11^, ^12^, ^13^, ^14^, ^15^, ^16^

As prehospital critical teams have expanded their scope of practice in the last ten years, there is a need to update a review of the current published literature to inform future practice. This systematic review aimed to assess the clinical outcomes of prehospital critical care compared to advanced life support for patients with out-of-hospital cardiac arrest and address the following specific research questions:

a) What is the composition of prehospital critical care teams attending out-of-hospital cardiac arrest patients?
b) Which out-of-hospital cardiac arrest patients are being attended by prehospital critical care teams?
c) What interventions are provided by prehospital critical care teams for out-of-hospital cardiac arrest patients?
d) What is the clinical effectiveness of prehospital critical care for out-of-hospital cardiac arrest patients?

## Methods

This systematic review was prospectively registered with PROSPERO (CRD42023478216) and its reporting follows the PRISMA 2020 statement (Supplementary File 1).^17^

### Eligibility criteria

The inclusion criteria were determined using the PICOST framework for ILCOR systematic reviews.^18^ The population was adults and children with attempted resuscitation after OHCA. The intervention was attendance of a CCT, defined as any provider with enhanced clinical competencies beyond that of standard paramedics using ALS algorithms and dedicated dispatch to critically ill patients. The comparator was ALS care by a non-critical care prehospital provider. Results will be reported as CCT and ALS groups in this review. The outcomes predefined as critical were favourable neurological outcome (defined as Cerebral Performance Category 1 or 2, modified Rankin Scale 0–3) at both hospital discharge, and at 30 days, survival to hospital discharge, survival at 30 days, and survival to hospital admission/return of spontaneous circulation. Eligible study designs were randomised controlled trials and non-randomised studies (non-randomised controlled trials, interrupted time series, before-and-after studies, cohort studies, registry-based studies), systematic reviews and meta-analyses. Unpublished studies (e.g., conference abstracts, trial protocols) were excluded. All relevant publications in any language were included, provided there was an English abstract. The timeframe was all years from database inception to 20 April 2024.

### Information sources and search strategy

Database searches of MEDLINE and Embase were undertaken using OVID. The CINAHL database was searched directly and the reference lists of eligible systematic reviews were reviewed for additional publications. The last search was undertaken on 20 April 2024.

The search strategy utilised a variety of MeSH terms and multipurpose terms, and terms were exploded or focussed as appropriate. These were combined with Boolean operators to expand and then refine the search. No search filters or limits were applied. The search strategies are included in Supplementary File 2 and was developed in collaboration with an information specialist at the University of Warwick, UK. Duplicates were removed using EndNote following a hierarchy of duplicate finding settings.^19^

### Study selection process

The titles and abstracts of returned articles were reviewed independently by at least two authors (AJB, RE, AG) against the eligibility criteria and the full texts of articles of interest obtained. The full texts were then assessed independently by two authors (AJB, RE) against the eligibility criteria and any discrepancies were resolved by discussion or involvement of an adjudicating author (MAS, JY).

### Data collection process and data items

Data were extracted independently by at least two authors (AJB, RE, AG) into a piloted and standardised template, as determined by the pre-registered protocol. The study-level variables included author and publication year, country of origin, study design, study time period, and EMS system description. The patient-level variables followed the Utstein template and included demographics (age and gender), witnessed status, Utstein location, bystander response (CPR and AED), first monitored rhythm, aetiology, and EMS response time.^20^ Descriptive data was extracted to answer the first three research questions, including descriptions of CCT composition (e.g. professional background, clinical competencies), description of patients attended and not attended by CCTs, and description of interventions delivered by CCTs. The outcomes sought were survival (at 30 days or hospital discharge), favourable neurological outcome (at 30 days or hospital discharge), and survival to hospital admission (survival to hospital admission or sustained prehospital ROSC). Favourable neurological outcome was defined as Cerebral Performance Category 1–2 or modified Rankin Scale 0–3. The measures of these dichotomous outcomes sought were summary statistics (absolute numbers and proportions) and relative effect measures (e.g. odds ratio). Where adjusted statistical analyses were performed, the variables used for adjustment were recorded.

### Synthesis methods

Synthesis was informed by the Cochrane Handbook for Systematic Reviews of Interventions.^21^ Study characteristics were firstly tabulated, allowing comparisons to be made and a synthesis of characteristics produced. The studies were grouped by aetiology (adult non-traumatic, traumatic, paediatric). A meta-analysis of each dichotomous outcome was then performed. Where studies reported unadjusted and adjusted analyses, the adjusted analyses were used. Some studies reported multiple adjusted analyses and the analysis judged to minimise the risk of confounding was selected for meta-analysis. Where standard errors were not reported by studies, they were calculated from confidence intervals and p values.^11^ Studies presenting unadjusted analyses at serious risk of bias were excluded from meta-analyses.^21^ Meta-analyses used the generic inverse-variance method with a random-effects approach given the use of non-randomised studies and risk of heterogeneity. The Chi^2^ and I^2^ statistics were used to quantify heterogeneity. RevMan online software was used for meta-analyses and to produce forest plot visualisations.^22^ Data extracted from included studies and data used for all analyses are available from the authors on reasonable request.^17^

### Risk of bias assessments

Individual study risk of bias was assessed independently by two authors (AJB and DC), with disagreements resolved by discussion and involvement of a third author (JY). All included studies were non-randomised studies of interventions and hence the ROBINS-I tool was used.^23^ Risk of bias plots were generated using the *robvis* R package.^24^

### Certainty assessment

The certainty of the body of evidence for each outcome was assessed using the Grading of Recommendations Assessment, Development and Evaluation (GRADE) approach, facilitated by GRADEPro software (Supplementary File 3).^25^, ^26^

## Results

### Study selection

**Fig. 1:**
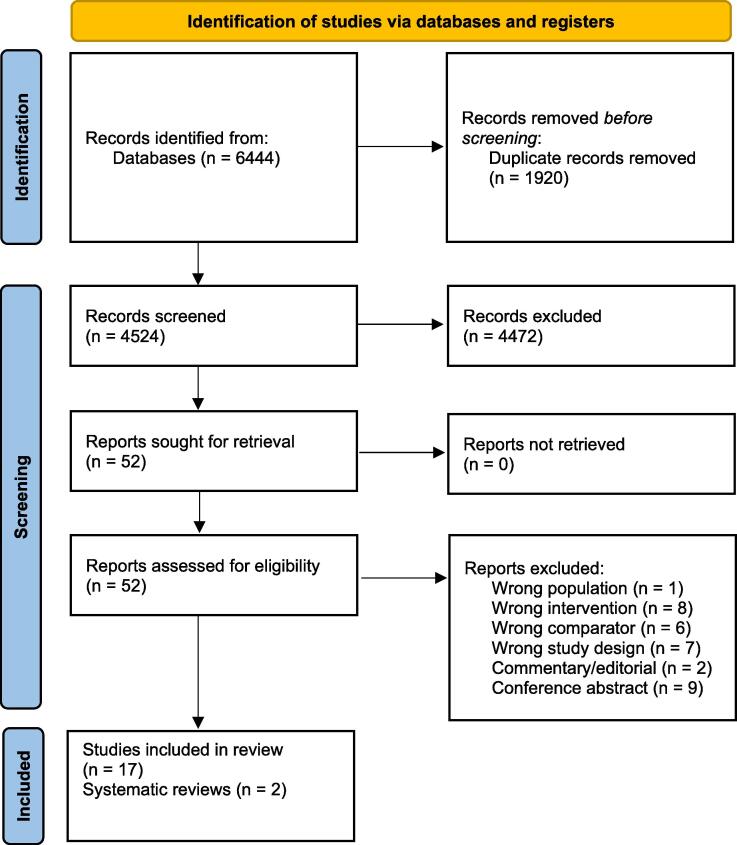
PRISMA diagram.

The search retrieved 6,444 results, and after duplicates were removed 4,524 records remained. Title and abstract screening identified 52 articles of interest and the full texts were obtained. Thirty-three articles were excluded after full text assessment for the reasons described in Fig. 1. A total of 17 original articles met eligibility criteria and were included.^15^, ^16^, ^10^, ^11^, ^12^, ^13^, ^27^, ^28^, ^29^, ^30^, ^31^, ^32^, ^33^, ^34^, ^35^, ^36^, ^37^ Two systematic reviews met eligibility criteria and their reference lists were reviewed, identifying no further studies for inclusion.^6^, ^9^

**Fig. 2:**
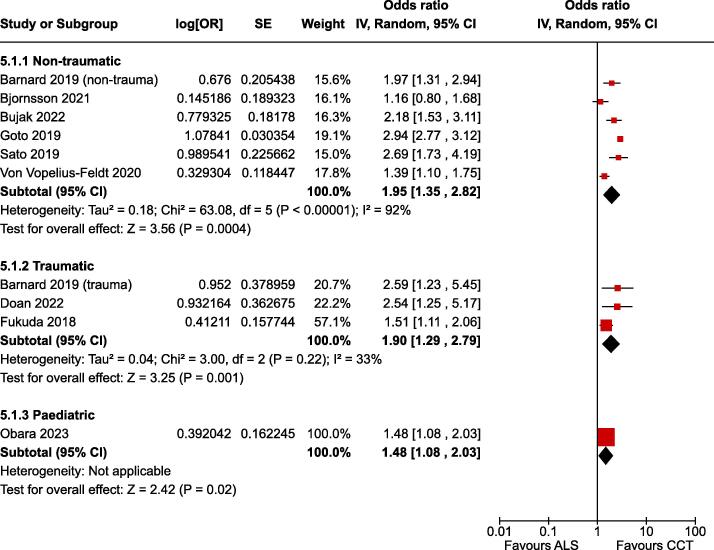
Forest plot of survival to hospital admission.

### Study characteristics

Study characteristics are summarised in Table 1. All studies were non-randomised. Ten studies used data from prospectively collected national registries,^10^, ^12^, ^15^, ^35^, ^37^, ^29^, ^30^, ^31^, ^32^, ^33^ four studies used prospective regional or ambulance service registries,^13^, ^16^, ^28^, ^34^ and three undertook case note reviews at a single ambulance service.^11^, ^27^, ^36^ One study used a before and after design following a change in EMS system,^12^ one study was a prospectively conducted cohort study,^10^ and the remainder were retrospective cohort studies (n = 15). All studies were undertaken in developed healthcare systems with the majority of studies undertaken in Japan (n = 8),^15^, ^35^, ^37^, ^29^, ^30^, ^31^, ^32^, ^33^ three in the United Kingdom,^10^, ^11^, ^36^ two in Australia,^16^, ^28^ and one each from Iceland,^12^ Poland,^13^ Norway^34^ and the USA.^27^ The majority of studies (n = 13) were published since 2015.^15^, ^16^, ^28^, ^29^, ^35^, ^36^, ^10^, ^11^, ^12^, ^13^, ^31^, ^32^, ^33^

**Table 1:** Study characteristics.

**Author and year**	**Country**	**Study design**	**Study time period**	**Sample size**	**Population**	**Description of prehospital critical care**
Barnard 2019^11^	UK	Retrospective cohort. Data from single ambulance service records.	1 January 2015 to 31 July 2017	9,109 (trauma 304, non-trauma 8,805)	All OHCA in which a resuscitation attempt made by the ambulance service.	Physician-paramedic staffed prehospital critical care team, dispatched by paramedic-led critical care desk at Emergency Operations Centre. Respond by helicopter or car.
Bjornsson 2021^12^	Iceland	Before and after. Data from prospective Utstein registry and hospital/coroner records.	2004–2007 (CCT) vs 2008–2014 (ALS)	471 (CCT 200, ALS 271)	OHCA with attempted resuscitation and considered to be cardiac aetiology.	Physician staffed ambulance. Specialists in emergency medicine or critical care.
Bujak 2022^13^	Poland	Retrospective cohort. Data from prospective Silesian registry of OHCA.	1 January 2018 to 31 December 2018	815 (CCT 351, ALS 461). PSM 351 in each group	Adult, EMS-treated, non-traumatic OHCA patients in Upper Silesia, Poland.	Physician staffed ambulance. Specialists in anaesthesia, emergency medicine, or intensive care.
Dickinson 1997^27^	USA	Retrospective cohort. Case note review at single ambulance service in New York, USA.	1 January 1994 to 31 December 1994	49 (CCT 9, ALS 40)	EMS-treated, non-traumatic OHCA. Excluded those presenting and remaining in asystole.	Physician staffed ambulance. Emergency medicine residents.
Doan 2022^28^	Australia	Retrospective cohort. Data from state ambulance service prospective OHCA registry.	1 January 2007 to 31 December 2019	1,148 (CCT 503, ALS 645)	Adult, EMS-treated traumatic cardiac arrest, secondary to blunt, penetrating or burn injury.	High acuity response unit (HARU) officers. Primarily critical care paramedics and occasionally physicians. Advanced skills in airway, blood transfusion, diagnostics, drugs, PHEA, surgical procedures.
Fukuda 2018^29^	Japan	Retrospective cohort. Data from prospective All-Japan Utstein registry.	1 January 2013 to 31 December 2014	2,419 (CCT 828, ALS 1,591). PSM 814 in each group	Traumatic OHCA following traffic collision and transported to hospital.	Physician staffed ambulance. Commonly specialists in emergency medicine with skills in blood transfusion and surgical procedures.
Goto 2013^30^	Japan	Retrospective cohort. Data from prospective All-Japan Utstein registry.	1 January 2007 to 31 December 2010	398,121	Adult, EMS-treated OHCA without prehospital ROSC.	Physician staffed ambulance.
Goto 2019^31^	Japan	Retrospective cohort. Data from prospective All-Japan Utstein registry.	1 January 2011 to 31 December 2015	613,251 (CCT 19,551, ALS 593,700) PSM 16,1612 in each group	EMS-treated OHCA.	Physician staffed ambulance. Specialists in emergency medicine or critical care with advanced skills in airway, blood transfusion, drugs, pacing and surgical procedures.
Hatakeyama 2021^15^	Japan	Retrospective cohort. Data from prospective Japanese Association for Acute Medicine registry.	1 June 2014 to 31 December 2017	19,247 (CCT 2,186, ALS 17,061) PSM 2,186 in each group	Adult, EMS-treated OHCA of medical aetiology, transported to hospital.	Prehospital physician in ambulance from scene to hospital arrival, by land ambulance or helicopter.
Nakajima 2023^32^	Japan	Retrospective cohort. Data from prospective Japanese Association for Acute Medicine registry.	1 June 2014 to 31 December 2019	1,269 (CCT 316, ALS 953)	Adult OHCA, medical aetiology, and received ECPR.	Physicians responding by ground ambulance or helicopter, mainly from tertiary emergency centres.
Obara 2023^33^	Japan	Retrospective cohort. Data from prospective Japanese Association for Acute Medicine registry.	1 June 2014 to 31 December 2019	1,187 (CCT 276, ALS 911)	Paediatric (age 17 and younger) OHCA.	Physicians responding by ground ambulance or helicopter. Experienced emergency physicians.
Olasveengen 2009^34^	Norway	Retrospective cohort. Data from prospective registry at ambulance service in Oslo.	1 May 2003 to 28 April 2008	973 (CCT 232, ALS 741)	Adult non-traumatic OHCA.	Physician staffed ambulance arriving as first resource. Specialists in anaesthesia (last year of training or board certified).
Pemberton 2023^16^	Australia	Retrospective cohort. Data from prospective Queensland ambulance service OHCA registry.	1 January 2002 to 31 December 2014	15,142 (CCT 9,838, ALS 5,304)	Adult, EMS-treated OHCA of presumed cardiac aetiology.	Critical care paramedics with skills in additional skills in advanced airway, critical care drugs and procedures.
Sato 2019^35^	Japan	Retrospective cohort. Data from prospective All-Japan Utstein registry.	1 January 2012 to 31 December 2016	892 (CCT 135, ALS 757)	Adult, bystander witnessed OHCA of medical aetiology in Niigata City, Japan.	Physician staffed ambulance. Specialists in emergency medicine or intensive care.
Von Vopelius-Feldt 2015^36^	UK	Retrospective cohort. Data from single ambulance service records.	April 2011 to April 2013	1,851 (CCT 165, ALS 1,686)	Adult, EMS-treated, non-traumatic OHCA.	Prehospital critical care team of prehospital physicians and specially trained critical care paramedics, responding by helicopter or rapid response vehicle. Both followed specific professional training programmes and had advanced skills in airway, drugs, helicopter transport, PHEA, surgical procedures.
Von Vopelius-Feldt 2020^10^	UK	Prospective cohort. Data from two neighbouring ambulance service OHCAO registry submission and local prehospital critical care teams.	September 2016 to October 2017	8,015 (CCT 866, ALS 7,149) PSM CCT 866, ALS 1847	Adult, EMS-treated, non-traumatic OHCA.	Prehospital critical care team of prehospital physicians and critical care paramedics, responding by helicopter or rapid response vehicle. Both followed specific professional training programmes. Physicians were senior specialists in emergency medicine, anaesthesia and/or intensive care.
Yasunaga 2010 ^37^	Japan	Retrospective cohort. Data from prospective All-Japan Utstein registry.	1 January 2005 to 31 December 2007	118,199 (CCT 4,509, ALS 113,690) In analysis CCT 1,597, ALS 53,482	EMS-treated, witnessed OHCA transported to hospital. For CCT analyses is patients without bystander CPR.	Physician with advanced skills in airway, anaesthesia, central venous access, inotropes, thrombolysis.

**Table 2:** Summary of review key findings. ALS: Advanced Life Support; IV: Intravenous; mCPR: Mechanical CPR; ROSC: Return of spontaneous circulation; RSI: Rapid sequence induction.

**Description of prehospital critical care teams**	• Nearly all studies included physicians (n=16) • Physician professional background was emergency medicine, anaesthesia, or intensive care • Five studies included critical care paramedics, all from UK (n=3) or Australia (n=2) • One study included only critical care paramedics • All teams could be transported by land, with six also being transported by helicopter
**Description of patients attended by prehospital critical care teams**	• Five studies reported significant differences in baseline characteristics between patients attended and not attended • In studies where differences were present, prehospital critical care patients had improved prognostic markers, including age, witnessed status, public setting, initial shockable rhythm, bystander CPR, and bystander defibrillation
**Interventions provided by prehospital critical care teams**	• Five studies reported interventions delivered, four of which made comparisons to the non-critical care group • The interventions more frequent with prehospital critical care were endotracheal intubation, adrenaline administration, amiodarone administration • One study reported greater IV cannula placement and reduced time to adrenaline with prehospital critical care • One study reported improved CPR metrics with prehospital critical care. This study also reported prehospital critical care cases had more personnel initially on scene (3-4 vs 2) • One study reported time to termination of CPR was reduced with prehospital critical care • One study reported in detail the enhanced interventions provided by prehospital critical care. During cardiac arrest, the most common interventions were mCPR, termination of resuscitation outside of standard guidelines, ultrasound use, IV drugs therapies outside of ALS. Following ROSC, the most common interventions were RSI, IV inotropes/vasopressors, sedation and/or paralysis that wasn’t RSI, bypass nearest hospital for cardiac arrest centre
**Favourable neurological outcome**	Adult non-traumatic: • Discharge: Very low certainty evidence from one study of 973 patients. OR 1.35 (0.71-2.60) • 30 days: Low certainty of evidence from six studies of 689,738 patients. OR 1.48 (1.19-1.84) Traumatic: • 30 days: Very low certainty evidence from one study of 2,419 patients. OR 3.76 (1.14-14.51) Paediatric: • 30 days: Very low certainty evidence from one study of 1,187 patients. OR 1.98 (1.08-3.66)
**Survival**	Adult non-traumatic: • Discharge: Low certainty evidence from seven studies of 12,171 patients. OR 1.34 (1.10-1.63) • 30 days: Low certainty of evidence from seven studies of 704,880 patients. OR 1.56 (1.38-1.75) Traumatic: • Discharge: Very low certainty evidence from two studies of 1,452 patients. OR 1.89 (0.94-3.84) • 30 days: Very low certainty evidence from one study of 2,419 patients. OR 3.76 (1.14-14.51) Paediatric: • 30 days: Very low certainty evidence from one study of 1,187 patients. OR 1.49 (0.97-2.88)
**Survival to hospital admission**	Adult non-traumatic: • Low certainty evidence from eight studies of 639,760 patients. OR 1.95 (1.35-2.82) Traumatic: • Very low certainty evidence from three studies of 2,419 patients. OR 1.90 (1.29-2.79) Paediatric: • Very low certainty evidence from one study of 1,187 patients. OR 1.48 (1.08-2.04)

A total of 1,192,158 patients were included, of which 3,871 were traumatic, 1,187 were paediatric, and the remainder were adult non-traumatic cardiac arrest. Two studies included only traumatic OHCA patients,^28^, ^29^ and one study reported traumatic and non-traumatic patients in separate analyses.^11^ One study included only paediatric patients.^33^ One study included only patients transported to hospital without prehospital ROSC,^30^ and one study included only patients going on to receive extracorporeal CPR.^32^ The majority of studies included adult, EMS-treated, non-traumatic OHCA patients.

### Descriptions of prehospital critical care teams

The descriptions of CCTs are summarised in Table 1. The majority of studies reported that prehospital critical care teams included physicians (n = 16). Where professional background was reported, these physicians were specialists in emergency medicine,^10^, ^12^, ^13^, ^27^, ^29^, ^31^, ^33^, ^35^ anaesthesia,^10^, ^13^, ^34^ or critical/intensive care.^10^, ^12^, ^13^, ^31^, ^35^ Five studies reported CCTs that included specially trained critical care paramedics.^10^, ^11^, ^16^, ^28^, ^36^ These studies were from the United Kingdom (n = 3)^10^, ^11^, ^36^ and Australia (n = 2)^16^, ^28^ only. One study reported CCTs including solely critical care paramedics.^16^ All studies reported CCTs could be transported by land, and six reported teams could be transported by helicopter.^10^, ^11^, ^15^, ^32^, ^33^, ^36^

### Description of patients attended by prehospital critical care teams

Most studies (n = 13) described the patients cared for by CCT teams and ALS teams (Supplementary Table 1). The remainder reported characteristics for the total sample.^11^, ^16^, ^28^, ^30^ Five studies reported statistically significant differences in baseline characteristics between the CCT and ALS groups.^10^, ^13^, ^29^, ^35^, ^36^ In these instances the CCT group had improved prognostic markers, including being younger,^10^, ^35^, ^36^ greater proportion male,^10^ greater proportion witnessed,^10^ greater proportion in a public setting,^10^, ^36^ more initial shockable rhythms,^10^, ^35^, ^36^ less initial asystole rhythm,^10^, ^29^, ^36^ greater proportion cardiac aetiology,^10^ greater bystander CPR,^10^, ^35^, ^36^ and greater bystander defibrillation.^10^ Two studies found the CCT group had a significantly longer EMS response time^10^, ^13^ and two studies found the CCT group had a significantly shorter EMS response time^29^, ^35^ The differences between CCT patients and ALS patients were most pronounced in the two UK studies that reported CCT and ALS baseline characteristics.^10^, ^36^

### Interventions provided by prehospital critical care teams

Five studies reported the interventions provided by CCTs,^10^, ^13^, ^31^, ^34^, ^35^ with four making comparisons between the CCT and ALS groups (Supplementary Table 1). Endotracheal tube placement was significantly more frequent with CCT care,^13^, ^34^ as was adrenaline^31^, ^35^ and amiodarone administration.^13^, ^34^ One study reported that intravenous catheter placement was greater with CCT care (81.5 % vs 69.6 %, p = 0.005) and time to adrenaline administration was reduced (20 min vs 22 min, p = 0.029).^35^ Another study found that patients receiving CCT care had improved manual CPR metrics of hands-off ratio and pre-shock pause.^34^ The authors report the physician-staffed ambulances had more personnel on board, leading to more personnel initially on scene (3–4 vs 2). One study reported that the time to termination of CPR was reduced in the CCT group (32 min vs 37 min, p < 0.01).^13^ One study reported the enhanced care interventions provided by CCTs.^10^ During cardiac arrest, the most common interventions were mechanical CPR (n = 204, 39 %), termination of resuscitation outside of standard guidelines (n = 124, 24 %), ultrasound (n = 97, 19 %), and specialist intravenous drug therapies (magnesium (n = 31, 6 %), sodium bicarbonate (n = 27, 5 %), calcium chloride (n = 15, 3 %)). Two hundred and three patients (39 %) did not receive an enhanced intervention beyond ALS care. Following ROSC, the most common interventions were rapid sequence induction (n = 94, 31 %), intravenous inotropes/vasopressors (n = 94, 31 %), sedation and/or paralysis that wasn’t rapid sequence induction (n = 64, 21 %), ultrasound (n = 13, 4 %), other intravenous drug therapies (amiodarone (n = 9, 3 %), magnesium (n = 7, 2 %), sodium bicarbonate (n = 5, 2 %)), and facilitating bypass of the nearest hospital for a cardiac arrest centre hospital (25 %).

### Risk of bias in studies

Fifteen studies were at overall moderate risk of bias and two studies were at serious risk of bias (Supplementary Files 4 & 5).^27^, ^34^ No study was at critical risk of bias. All studies were at moderate or serious risk of bias due to confounding. Those at serious risk of bias due to confounding used unadjusted or incomplete adjusted analyses and were excluded from meta-analyses.

## Outcomes

The outcomes of individual studies are summarised in Supplementary Table 2.

### Favourable neurological outcome at hospital discharge

For the critical outcome of favourable neurological outcome at hospital discharge in adult non-traumatic OHCA there was very low certainty evidence (downgraded for risk of bias and imprecision) from one non-randomised study enrolling 973 patients, showing no significant difference (OR 1.35, 95% CI 0.71–2.60).^34^

There was no evidence in traumatic or paediatric OHCA to address the critical outcome of favourable neurological outcome at hospital discharge.

### Favourable neurological outcome at 30 days

**Fig. 3:**
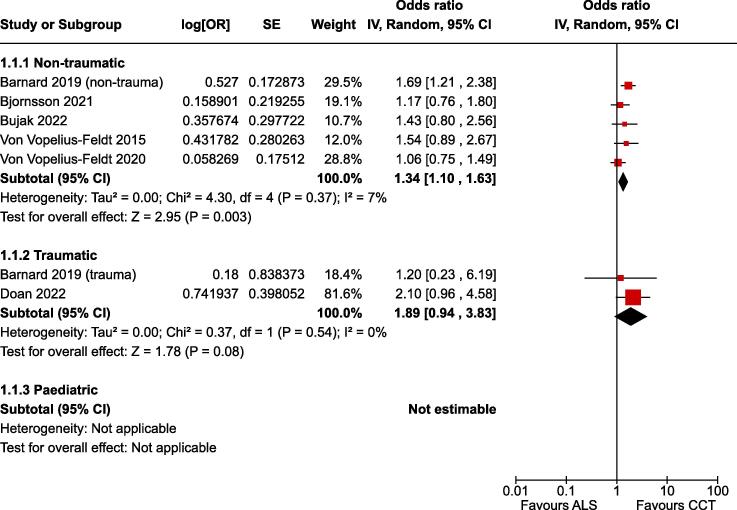
Forest plot of favourable neurological outcome at 30 days.

For the critical outcome of favourable neurological outcome at 30 days in adult non-traumatic OHCA there was low certainty evidence (downgraded for risk of bias) from six non-randomised studies enrolling 689,738 patients.^15^, ^35^, ^37^, ^30^, ^31^, ^32^ The estimate of effect indicated improved outcome associated with CCTs (OR 1.48, 95% CI 1.19–1.84) (Fig. 3).

For the critical outcome of favourable neurological outcome at 30 days in traumatic OHCA there was very low certainty evidence (downgraded for risk of bias and imprecision) from one non-randomised study enrolling 2,419 patients.^29^ The estimate of effect indicated improved outcome associated with CCTs (OR 3.76, 95% CI 1.14–14.51) (Fig. 3).

For the critical outcome of favourable neurological outcome at 30 days in paediatric OHCA there was very low certainty evidence (downgraded for risk of bias and imprecision) from one non-randomised study enrolling 1,187 patients.^33^ The estimate of effect indicated improved outcome associated with CCTs (OR 1.98, 95% CI 1.08–3.66) (Fig. 3).

### Survival to hospital discharge

**Fig. 4:**
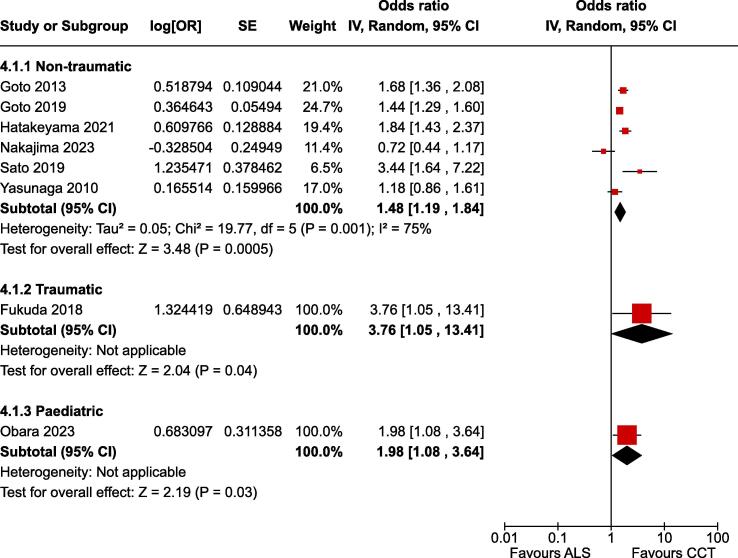
Forest plot of survival to hospital discharge.

For the critical outcome of survival to hospital discharge in adult non-traumatic OHCA there was low certainty evidence (downgraded for risk of bias) from seven non-randomised studies enrolling 12,171 patients.^27^, ^34^, ^36^, ^10^, ^11^, ^12^, ^13^ The estimate of effect indicated improved outcome associated with CCTs (OR 1.34, 95% CI 1.10–1.63) (Fig. 4).

For the critical outcome of survival to hospital discharge in traumatic OHCA there was very low certainty evidence (downgraded for risk of bias and imprecision) from two non-randomised studies enrolling 1,452 patients.^11^, ^28^ The effect estimate suggested improved outcome associated with CCTs, however this finding was not statistically significance at the 5 % level (OR 1.89, 95% CI 0.94–3.84) (Fig. 4).

There was no evidence in paediatric OHCA to address the critical outcome of survival to hospital discharge.

### Survival at 30 days

**Fig. 5:**
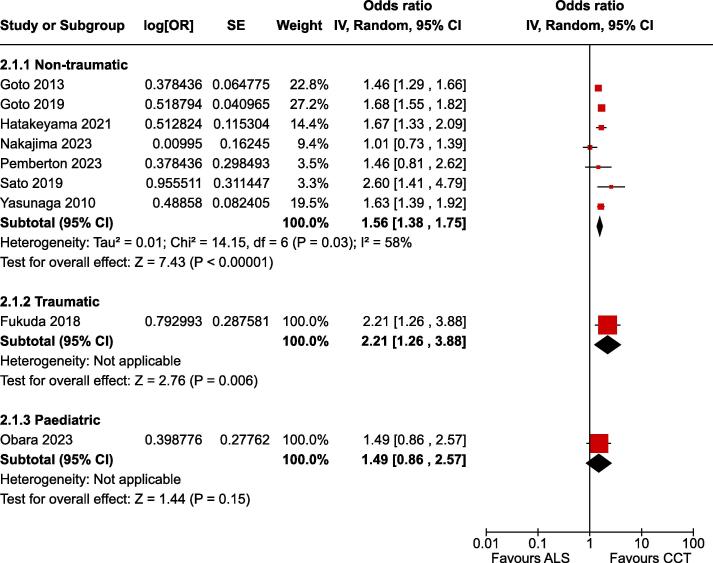
Forest plot of survival at 30 days.

For the critical outcome of survival at 30 days in adult non-traumatic OHCA there was low certainty evidence (downgraded for risk of bias) from seven non-randomised studies enrolling 704,880 patients.^15^, ^16^, ^35^, ^37^, ^30^, ^31^, ^32^ The estimate of effect indicated improved outcome associated with CCTs (OR 1.56, 95% CI 1.38–1.75) (Fig. 5).

For the critical outcome of survival at 30 days in traumatic OHCA there was very low certainty evidence (downgraded for risk of bias and imprecision) from one non-randomised study enrolling 2,419 patients.^29^ The estimate of effect indicated improved outcome associated with CCTs (OR 2.21, 95% CI 1.26–3.89) (Fig. 5).

For the critical outcome of survival at 30 days in paediatric OHCA there was very low certainty evidence (downgraded for risk of bias and imprecision) from one non-randomised study enrolling 1,187 patients.^33^ The effect estimate suggested improved outcome associated with CCTs, however this finding was not statistically significance at the 5 % level (OR 1.49, 95% CI 0.97–2.88) (Fig. 5).

### Survival to hospital admission

For the critical outcome of survival to hospital admission in adult non-traumatic OHCA there was low certainty evidence (downgraded for risk of bias) from eight non-randomised studies enrolling 639,760 patients.^27^, ^31^, ^34^, ^35^, ^10^, ^11^, ^12^, ^13^ The estimate of effect indicated improved outcome associated with CCTs (OR 1.95, 95% CI 1.35–2.82) (Fig. 2).

For the critical outcome of survival to hospital admission in traumatic OHCA there was very low certainty evidence (downgraded for risk of bias and imprecision) from three non-randomised studies enrolling 2,419 patients.^11^, ^28^, ^29^ The estimate of effect indicated improved outcome associated with CCTs (OR 1.90, 95% CI 1.29–2.79) (Fig. 2).

For the critical outcome of survival to hospital admission in paediatric OHCA there was very low certainty evidence (downgraded for risk of bias and imprecision) from one non-randomised study enrolling 1,187 patients.^33^ The estimate of effect indicated improved outcome associated with CCTs (OR 1.48, 95% CI 1.08–2.04) (Fig. 2).

## Discussion

This systematic review and meta-analysis of CCTs for OHCA contains over 1.1 million patients and includes traumatic and paediatric cardiac arrest subgroups for the first time. A comparison has been made with ALS to reflect current guidelines and clinical practice. There was a consistent signal of benefit for CCTs across all outcomes and subgroups. Meta-analysis revealed that CCTs were associated with improvement in survival to hospital admission, survival to hospital discharge/30 days, and favourable neurological outcome. The certainty of evidence for these outcomes in adult non-traumatic cardiac arrests was low, and for traumatic and paediatric cardiac arrests was very low. CCTs included prehospital physicians in almost all studies. Critical care paramedics were part of CCTs in studies from Australia and the United Kingdom only. Most of the evidence is from national registries in Japan, followed by Europe and Australia. There is extremely limited evidence from North America and no evidence from less developed healthcare systems.

No randomised studies were retrieved and hence caution is needed. All studies included in meta-analyses were at moderate risk of bias, principally due to confounding. CCT resources may be targeted to OHCAs with a better prognosis. Five of the included studies reported that the CCT attended OHCAs had more favourable baseline characteristics, and this was most evident in the UK studies, possibly reflecting differences in EMS systems. Although there was a consistent signal of benefit with CCTs, these observational findings may not be replicated in any future randomised study, as has recently been highlighted with cardiac arrest centre hospitals and the ARREST trial.^38^, ^39^, ^40^ However, there are multiple barriers to undertaking a randomised trial in this setting and higher quality evidence may not be produced. A study of key stakeholders in the UK highlighted concerns regarding randomising patients to CCT attendance due to a lack of equipoise.^41^ Patient and public groups, air ambulance charities, and CCT providers were uncomfortable with randomisation due to perceived benefits of CCTs, as well as concerns that randomisation could conflict with air ambulance charities’ objectives and threaten the professional identify of prehospital critical care providers.

Traumatic and paediatric cardiac arrests are groups where the experience and specialist capabilities of CCTs in delivering individualised care may be able offer significant benefit.^42^ There special circumstance arrests are emotive and cognitively challenging,^43^ may require complex and high risk interventions,^44^, ^45^ and exposure to these rarer events may be more limited for EMS teams.^46^ However, this review highlights the very low certainty of clinical evidence in these groups, with just one study examining paediatric cardiac arrest.

The signal of benefit was generally consistent across studies and outcomes. An exception was the study by Nakajima *et al.*, the only study to report any effect estimates not favouring CCTs.^32^ This study’s patient population was unique since it only included OHCA patients who underwent extracorporeal CPR. These patients had a relatively high 1-month survival and favourable neurological outcome of 20 % and 10 % respectively. The effect estimate for neurological outcome favoured no prehospital physician presence (adjusted odds ratio 0.72), although the confidence intervals crossed one (95% CI 0.44–1.17). Prehospital physician presence did not result in a reduced time to extracorporeal CPR initiation (median time from call 57 min), likely explaining the failure to produce benefit.

We were unable to discern what interventions CCTs were provided to OHCA patients by CCTs, with just one study reporting individual enhanced interventions, and hence the mechanisms underpinning improvement in outcomes is unclear. Some of these enhanced interventions have not demonstrated clinical superiority when tested in pragmatic randomised trials.^47^, ^48^ It may be that clinical benefits are due to better delivery of ALS care as opposed to specialist interventions. One study reported that 39 % of patients attended by a CCT did not receive enhanced interventions beyond ALS.^10^ Some studies in this review reported that CCTs had greater rates of intravenous catheter placement,^35^ adrenaline and amiodarone administration,^13^, ^30^, ^34^, ^35^ reduced time to adrenaline delivery,^35^ and improved manual CPR quality metrics.^34^ This provides an indication of ALS standards of practice in the comparator group, which may influence the comparative effectiveness of CCTs. OHCAs attended by CCTs may have more personnel on scene, either due to more crew in the CCT,^34^ or because CCTs back up ALS teams,^10^ therefore facilitating higher quality ALS standard care. The greater exposure to cardiac arrest, experience, crew resource management skills, and training of CCTs may contribute to more effective delivery of ALS.

The benefits of CCTs may be most marked in the post-ROSC patient, where delivery of more personalised post resuscitation care, including invasive blood pressure monitoring, cardiovascular support and prehospital emergency anaesthesia, may be beyond the scope and expertise of other EMS personnel.^5^, ^42^ This hypothesis remains unaddressed by the present literature as the review identified no study or subgroup analysis investigating post-ROSC patients. One study reported that CCTs were frequently bypassing nearer hospitals in favour of cardiac arrest centres.^10^ Direct transport to a cardiac arrest centre has been associated with improved survival, and hence CCT facilitated transfer to more optimal hospital care may lead to improved outcomes.^38^, ^40^, ^49^ An important intervention by CCTs in some EMS systems is the ability to make advanced decisions to terminate a resuscitation attempt outside of standard guidelines. A UK study reported that nearly a quarter of patients attended in-arrest by a CCT had the resuscitation attempt terminated.^10^ Whilst this will not improve clinical outcomes, it offers multiple benefits including prioritising patient dignity by minimising delivery of aggressive and futile care, alleviating distress amongst healthcare professionals and bystanders,^50^, ^51^ preventing risky in-arrest emergency transport,^52^, ^53^ reducing healthcare system and hospital demands,^54^ and preventing separation of patients from grieving relatives.^55^, ^56^

Decisions regarding implementation and expansion of CCTs for OHCA patients will be setting specific. The present literature is dominated by studies from Japan, and hence this may affect generalisability of findings. In contrast to other included countries, Japanese EMS teams will immediately provide basic life support and deliver ALS once authorisation is received from an on-call physician. Nevertheless, the benefit of CCTs was consistent and also present in studies from Europe and Australia. The clinical effectiveness of CCTs is likely to be influenced by the EMS system within which they are deployed, the standard of the CCT, and the standard of the comparator group. This review has compared CCT with ALS care since this represents current guidelines and clinical practice.^7^, ^8^ A previous review compared physician-guided resuscitation with paramedic-guided resuscitation, including basic life support in the paramedic-guided control group, and found that physician-guided care was associated with improved outcomes.^6^ Introducing and expanding CCT services will present resource implications. The costs and complexities will also be system specific. A study from the UK reported that CCTs incurred greater monetary cost than paramedic-delivered ALS care.^57^ The authors calculated that to be cost-effective at a willingness to pay threshold of £20000 per quality-adjusted life year, the minimally economically important difference was a 3–5 % absolute improvement in survival to hospital discharge with CCT care. The main driver of this cost was the helicopter, however CCTs also respond by rapid response vehicle in some settings, emphasising the system specific nature of implementation decisions.

CCTs incur greater cost than other EMS teams and are a scarce resource that once deployed are unable to attend concurrent incidents.^57^, ^58^ This review demonstrates that in some EMS systems CCTs attend OHCA patients with improved prognostic markers. The approach to CCT dispatch varies between systems, with some using non-clinician tasking algorithms and others using expert clinician assessments within an interrogated call model.^58^, ^59^, ^60^, ^61^ CCT dispatch will also be dependent on the standards and interactions of other services within the system. This variation highlights that ensuring appropriate tasking is a challenge and priority for many EMS systems.^62^, ^63^ There is an ongoing need to better understand which patients would benefit most from CCTs and optimise CCT dispatch.^64^

International evidence continues to mount of health inequalities for cardiac arrest patients, including in the EMS response.^65^, ^66^, ^67^, ^68^, ^69^, ^70^ Tackling these disparities is a priority for resuscitation organisations.^71^ The effect CCTs have on inequalities remains unclear. Given the possible clinical efficacy demonstrated in this review, disparities in access to CCT care could contribute to inequalities. Conversely, CCTs may help address inequalities by more readily reaching disadvantaged patients. Optimisation of the EMS response, including provision of CCT care, alongside targeted CPR training and public access defibrillator placement could be part of a wider strategy to tackle inequalities.^72^, ^73^, ^74^

The review has underlined the value of OHCA registries. Almost all included studies used an OHCA registry as the data source and over 1.1 million patients are included in this review. There has been an expansion in OHCA registries over recent years and this is reflected in over 663,000 of the included patients being reported in registry-based studies published since the previous review in 2016.^9^ Routine collection of individual OHCA information in registries is allowing benchmarking and supporting quality improvement, as well as facilitating large observational studies such as those included within this review.^75^

This review is principally limited by the non-randomised nature of all included studies, all of which were at moderate or severe risk of bias. This is reflected in the certainty of evidence assessments. There was a paucity of evidence for paediatric and traumatic cardiac arrests, including 1,187 and 3,871 patients from one and three studies respectively. The certainty of evidence in these groups was downgraded to very low due to risk of bias and imprecision. There was heterogeneity in EMS systems and patient characteristics, with one study including patients without prehospital ROSC and another including patients who went on to receive extracorporeal CPR.^30^, ^32^ This may bias meta-analysis, and therefore a generic inverse-variance approach with a random-effects was used. Heterogeneity was present in CCTs, with studies inconsistently reporting the specific competencies of teams and the equipment carried. There is a risk of publication bias, however funnel plots did not reveal convincing evidence of bias (Supplementary File 6). Finally, although the signal of benefit was present across EMS systems, it should be acknowledged that most patients were reported by studies from Japan.

## Conclusions

Prehospital critical care is associated with improved outcomes for out-of-hospital cardiac arrest patients. There is low certainty of evidence for adult non-traumatic cardiac arrest and very low certainty of evidence for traumatic and paediatric cardiac arrest. Prehospital critical care teams are treating cardiac arrest patients across multiple international EMS systems and meta-analysis has revealed improvements in survival to hospital admission, survival to hospital discharge/30 days, and favourable neurological outcome. Implementation and expansion of these teams is likely to incur significant resource, training, and EMS infrastructure costs that are setting specific. The patient groups most likely to benefit from prehospital critical care remain unclear and there is a paucity of evidence examining paediatric and traumatic cardiac arrests, key subgroups where specialist individualised care may offer benefit.

## CRediT authorship contribution statement

**Adam J Boulton:** Writing – review & editing, Writing – original draft, Visualization, Validation, Methodology, Investigation, Formal analysis, Conceptualization. **Rachel Edwards:** Writing – review & editing, Investigation, Conceptualization. **Andrew Gadie:** Writing – review & editing, Investigation, Conceptualization. **Daniel Clayton:** Writing – review & editing, Investigation, Conceptualization. **Caroline Leech:** Writing – review & editing, Supervision, Methodology, Conceptualization. **Michael A. Smyth:** Writing – review & editing, Supervision, Methodology, Investigation, Conceptualization. **Terry Brown:** Writing – review & editing, Methodology, Conceptualization. **Joyce Yeung:** Writing – review & editing, Supervision, Methodology, Investigation, Conceptualization.

## Declaration of competing interest

The authors declare the following financial interests/personal relationships which may be considered as potential competing interests: Adam J Boulton (Doctoral Research Fellow, NIHR303023) is funded by the NIHR. The views expressed in this publication are those of the author(s) and not necessarily those of the NIHR, NHS or the UK Department of Health and Social Care.

## Figures and Tables

**Supplementary Fig. 1:**
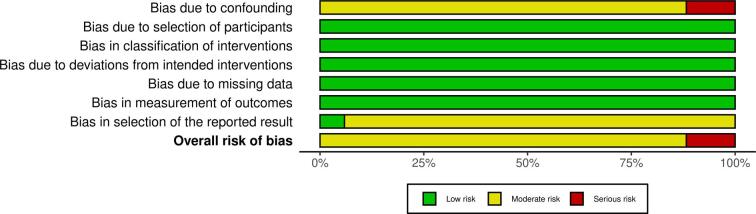


**Supplementary Fig. 2:**
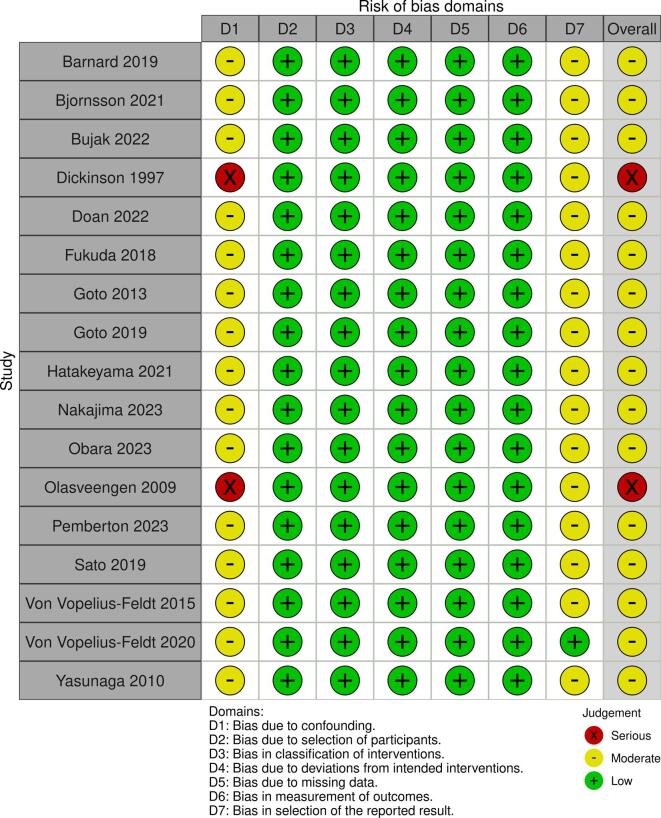


## Appendix A

The following ILCOR EIT Task Force members are acknowledged as collaborators on this systematic review: Cristian Abelairas-Gómez, Natalie Anderson, Jan Breckwoldt, Adam Cheng, Andrea Cortegiani, Aaron Donoghue, Kathryn Eastwood, Barbara Farquharson, Robert Greif, Ming-Ju Hsieh, Ying-Chih Ko, Kasper G. Lauridsen, Jeffrey Lin, Andrew Lockey, Tasuku Matsuyama, Sabine Nabecker, Kevin Nation, Alexander Olaussen, Taylor L. Sawyer, Sebastian Schnaubelt, Chih-Wei Yang.
