# Supplementary material for: Prehospital critical care beyond advanced life support for out-of-hospital cardiac arrest: A systematic review
Source: Resusc Plus. 2024 Dec 12;21:100803. doi: 10.1016/j.resplu.2024.100803 (PMC11728073; doi:10.1016/j.resplu.2024.100803)
Supplement: Supplementary Data 2 [file mmc2.docx]

Ovid MEDLINE(R) <1946 to May Week 5 2024>

Embase <1974 to 2024 June 11>

1 prehospital physician*.mp.

2 pre-hospital physician*.mp.

3 prehospital clinician*.mp.

4 pre-hospital clinician*.mp.

5 prehospital care clinician*.mp.

6 pre-hospital care clinician*.mp.

7 prehospital EMS physician*.mp.

8 pre-hospital EMS physician*.mp.

9 prehospital nurse*.mp.

10 pre-hospital nurse*.mp.

11 prehospital critical care.mp.

12 pre-hospital critical care.mp.

13 critical care paramedic.mp.

14 exp Air Ambulances/

15 *Emergency Medical Services/

16 1 or 2 or 3 or 4 or 5 or 6 or 7 or 8 or 11 or 12 or 13 or 14 or 15

17 *Out-of-Hospital Cardiac Arrest/

18 *Cardiopulmonary Resuscitation/

19 17 or 18

20 16 and 19

CINAHL

(("prehospital physician*") OR ("pre-hospital physician*") OR ("prehospital clinician*") OR ("pre-hospital clinician*") OR ("prehospital care clinician*") OR ("pre-hospital care clinician*") OR ("prehospital EMS physician*") OR ("pre-hospital EMS physician*") OR ("prehospital critical care") OR ("pre-hospital critical care") OR ("critical care paramedic") OR ("air ambulance") OR ((MM "Emergency Medical Services"))) AND (("out-of-hospital cardiac arrest") OR ((MM "Heart Arrest")) OR ((MM "Resuscitation, Cardiopulmonary")))
