# Supplementary material for: Prehospital critical care beyond advanced life support for out-of-hospital cardiac arrest: A systematic review
Source: Resusc Plus. 2024 Dec 12;21:100803. doi: 10.1016/j.resplu.2024.100803 (PMC11728073; doi:10.1016/j.resplu.2024.100803)
Supplement: Supplementary Data 3 [file mmc3.docx]

**Author(s):** Adam J Boulton

**Question:** Prehospital critical care compared to advanced life support for patients with out-of-hospital cardiac arrest

**Setting:**

**Bibliography:**

| **Certainty assessment** | | | | | | | **№ of patients** | | **Effect** | | **Certainty** | **Importance** |
| --- | --- | --- | --- | --- | --- | --- | --- | --- | --- | --- | --- | --- |
| **№ of studies** | **Study design** | **Risk of bias** | **Inconsistency** | **Indirectness** | **Imprecision** | **Other considerations** | **prehospital critical care** | **advanced life support** | **Relative (95% CI)** | **Absolute (95% CI)** |  |  |

| **Survival to hospital admission / return of spontaneous circulation - non-trauma** | | | | | | | | | | | | |
| --- | --- | --- | --- | --- | --- | --- | --- | --- | --- | --- | --- | --- |
| 8 | non-randomised studies | serious^a^ | not serious | not serious | serious^b^ | none | 6035/31337 (19.3%) | 50789/608423 (8.3%) | **OR 1.95** (1.35 to 2.82) | **67 more per 1,000** (from 26 more to 121 more) | ⨁⨁◯◯ Low | CRITICAL |
| **Survival to hospital admission / return of spontaneous circulation - trauma** | | | | | | | | | | | | |
| 3 | non-randomised studies | serious^a^ | not serious | not serious | very serious^b^ | none | 94/828 (11.4%) | 110/1591 (6.9%) | **OR 1.90** (1.29 to 2.79) | **55 more per 1,000** (from 18 more to 103 more) | ⨁◯◯◯ Very low | CRITICAL |
| **Survival to hospital admission / return of spontaneous circulation - paediatric** | | | | | | | | | | | | |
| 1 | non-randomised studies | serious^a^ | not serious | not serious | very serious^e^ | none | 97/276 (35.1%) | 240/911 (26.3%) | **OR 1.48** (1.08 to 2.04) | **83 more per 1,000** (from 15 more to 158 more) | ⨁◯◯◯ Very low | CRITICAL |

| **Survival to hospital discharge - non-trauma** | | | | | | | | | | | | |
| --- | --- | --- | --- | --- | --- | --- | --- | --- | --- | --- | --- | --- |
| 7 | non-randomised studies | serious^a^ | not serious | not serious | serious^b^ | none | 252/1823 (13.8%) | 896/10348 (8.7%) | **OR 1.34** (1.10 to 1.63) | **26 more per 1,000** (from 8 more to 47 more) | ⨁⨁◯◯ Low | CRITICAL |
| **Survival to hospital discharge - trauma** | | | | | | | | | | | | |
| 2 | non-randomised studies | serious^a^ | not serious | not serious | very serious^c^ | none |  |  | **OR 1.89** (0.94 to 3.84) | **0 fewer per 1,000** (from 0 fewer to 0 fewer)^d^ | ⨁◯◯◯ Very low | CRITICAL |
| **Survival at 30 days - non-trauma** | | | | | | | | | | | | |
| 7 | non-randomised studies | serious^a^ | not serious | not serious | serious^b^ | none | 2824/33623 (8.4%) | 33585/671257 (5.0%) | **OR 1.56** (1.38 to 1.75) | **26 more per 1,000** (from 18 more to 34 more) | ⨁⨁◯◯ Low | CRITICAL |
| **Survival at 30 days - trauma** | | | | | | | | | | | | |
| 1 | non-randomised studies | serious^a^ | not serious | not serious | very serious^e^ | none | 26/828 (3.1%) | 25/1591 (1.6%) | **OR 2.21** (1.26 to 3.89) | **18 more per 1,000** (from 4 more to 43 more) | ⨁◯◯◯ Very low | CRITICAL |
| **Survival at 30 days - paediatric** | | | | | | | | | | | | |
| 1 | non-randomised studies | serious^a^ | not serious | not serious | very serious^e^ | none | 46/276 (16.7%) | 115/911 (12.6%) | **OR 1.49** (0.97 to 2.88) | **51 more per 1,000** (from 3 fewer to 168 more) | ⨁◯◯◯ Very low | CRITICAL |
| **Favourable neurological outcome at hospital discharge - non-trauma** | | | | | | | | | | | | |
| 1 | non-randomised studies | not serious^a^ | not serious | not serious | very serious^e^ | none | 29/232 (12.5%) | 75/741 (10.1%) | **OR 1.35** (0.71 to 2.60) | **31 more per 1,000** (from 27 fewer to 125 more) | ⨁⨁◯◯ Low | CRITICAL |
| **Favourable neurological outcome at 30 days - non-trauma** | | | | | | | | | | | | |
| 6 | non-randomised studies | serious^a^ | not serious | not serious | serious^b^ | none | 1496/23785 (6.3%) | 17146/665953 (2.6%) | **OR 1.48** (1.19 to 1.84) | **12 more per 1,000** (from 5 more to 21 more) | ⨁⨁◯◯ Low | CRITICAL |
| **Favourable neurological outcome at 30 days - trauma** | | | | | | | | | | | | |
| 1 | non-randomised studies | serious^a^ | not serious | not serious | very serious^e^ | none | 8/828 (1.0%) | 4/1591 (0.3%) | **OR 3.76** (1.14 to 14.51) | **7 more per 1,000** (from 0 fewer to 33 more) | ⨁◯◯◯ Very low | CRITICAL |
| **Favourable neurological outcome at 30 days - paediatric** | | | | | | | | | | | | |
| 1 | non-randomised studies | serious^a^ | not serious | not serious | very serious^e^ | none | 23/276 (8.3%) | 33/911 (3.6%) | **OR 1.98** (1.08 to 3.66) | **33 more per 1,000** (from 3 more to 85 more) | ⨁◯◯◯ Very low | CRITICAL |

**CI:** confidence interval; **OR:** odds ratio

#### Explanations

a. ROBINS-I tool assessment.

b. Some studies not reporting number of events or totals. Some studies imprecise effect estimates with wide confidence intervals.

c. Neither study reported number of events or totals. Imprecise effect estimates with wide confidence intervals.

d. Unable to calculate as number of events and totals are not reported.

e. Single study with wide confidence interval.
