# Supplementary material for: Prehospital critical care beyond advanced life support for out-of-hospital cardiac arrest: A systematic review
Source: Resusc Plus. 2024 Dec 12;21:100803. doi: 10.1016/j.resplu.2024.100803 (PMC11728073; doi:10.1016/j.resplu.2024.100803)
Supplement: Supplementary Data 4 [file mmc4.pdf]

Author(s): Adam J Boulton

Question: Prehospital critical care compared to advanced life support for patients with out-of-hospital cardiac arrest

Setting:

Bibliography:

Certainty assessment							№ of patients		Effect		Certainty	Importance
№ of studies	Study design	Risk of bias	Inconsistency	Indirectness	Imprecision	Other considerations	prehospital critical care	advanced life support	Relative (95% CI)	Absolute (95% CI)		
**Survival to hospital admission / return of spontaneous circulation - non-trauma**												
8	non-randomised studies	serious[a]	not serious	not serious	serious[b]	none	6035/31337 (19.3%)	50789/608423 (8.3%)	**OR 1.95** (1.35 to 2.82)	**67 more per 1,000** (from 26 more to 121 more)	⊕⊕◯◯ Low	CRITICAL
**Survival to hospital admission / return of spontaneous circulation - trauma**												
3	non-randomised studies	serious[a]	not serious	not serious	very serious[b]	none	94/828 (11.4%)	110/1591 (6.9%)	**OR 1.90** (1.29 to 2.79)	**55 more per 1,000** (from 18 more to 103 more)	⊕◯◯◯ Very low	CRITICAL
**Survival to hospital admission / return of spontaneous circulation - paediatric**												
1	non-randomised studies	serious[a]	not serious	not serious	very serious[e]	none	97/276 (35.1%)	240/911 (26.3%)	**OR 1.48** (1.08 to 2.04)	**83 more per 1,000** (from 15 more to 158 more)	⊕◯◯◯ Very low	CRITICAL
**Survival to hospital discharge - non-trauma**												
7	non-randomised studies	serious[a]	not serious	not serious	serious[b]	none	252/1823 (13.8%)	896/10348 (8.7%)	**OR 1.34** (1.10 to 1.63)	**26 more per 1,000** (from 8 more to 47 more)	⊕⊕◯◯ Low	CRITICAL
**Survival to hospital discharge - trauma**												
2	non-randomised studies	serious[a]	not serious	not serious	very serious[c]	none			**OR 1.89** (0.94 to 3.84)	**0 fewer per 1,000** (from 0 fewer to 0 fewer)[d]	⊕◯◯◯ Very low	CRITICAL
**Survival at 30 days - non-trauma**												
7	non-randomised studies	serious[a]	not serious	not serious	serious[b]	none	2824/33623 (8.4%)	33585/671257 (5.0%)	**OR 1.56** (1.38 to 1.75)	**26 more per 1,000** (from 18 more to 34 more)	⊕⊕◯◯ Low	CRITICAL
**Survival at 30 days - trauma**												

1	non-randomised studies	serious[a]	not serious	not serious	very serious[e]	none	26/828 (3.1%)	25/1591 (1.6%)	**OR 2.21** (1.26 to 3.89)	**18 more per 1,000** (from 4 more to 43 more)	⨁◯◯◯ Very low	CRITICAL
**Survival at 30 days - paediatric**												
1	non-randomised studies	serious[a]	not serious	not serious	very serious[e]	none	46/276 (16.7%)	115/911 (12.6%)	**OR 1.49** (0.97 to 2.88)	**51 more per 1,000** (from 3 fewer to 168 more)	⨁◯◯◯ Very low	CRITICAL
**Favourable neurological outcome at hospital discharge - non-trauma**												
1	non-randomised studies	not serious[a]	not serious	not serious	very serious[e]	none	29/232 (12.5%)	75/741 (10.1%)	**OR 1.35** (0.71 to 2.60)	**31 more per 1,000** (from 27 fewer to 125 more)	⨁⨁◯◯ Low	CRITICAL
**Favourable neurological outcome at 30 days - non-trauma**												
6	non-randomised studies	serious[a]	not serious	not serious	serious[b]	none	1496/23785 (6.3%)	17146/665953 (2.6%)	**OR 1.48** (1.19 to 1.84)	**12 more per 1,000** (from 5 more to 21 more)	⨁⨁◯◯ Low	CRITICAL
**Favourable neurological outcome at 30 days - trauma**												
1	non-randomised studies	serious[a]	not serious	not serious	very serious[e]	none	8/828 (1.0%)	4/1591 (0.3%)	**OR 3.76** (1.14 to 14.51)	**7 more per 1,000** (from 0 fewer to 33 more)	⨁◯◯◯ Very low	CRITICAL
**Favourable neurological outcome at 30 days - paediatric**												
1	non-randomised studies	serious[a]	not serious	not serious	very serious[e]	none	23/276 (8.3%)	33/911 (3.6%)	**OR 1.98** (1.08 to 3.66)	**33 more per 1,000** (from 3 more to 85 more)	⨁◯◯◯ Very low	CRITICAL

CI: confidence interval; **OR:** odds ratio

Explanations

a. ROBINS-I tool assessment.

b. Some studies not reporting number of events or totals. Some studies imprecise effect estimates with wide confidence intervals.

c. Neither study reported number of events or totals. Imprecise effect estimates with wide confidence intervals.

d. Unable to calculate as number of events and totals are not reported.

e. Single study with wide confidence interval.
