# Supplementary material for: Prehospital critical care beyond advanced life support for out-of-hospital cardiac arrest: A systematic review
Source: Resusc Plus. 2024 Dec 12;21:100803. doi: 10.1016/j.resplu.2024.100803 (PMC11728073; doi:10.1016/j.resplu.2024.100803)

ROSC

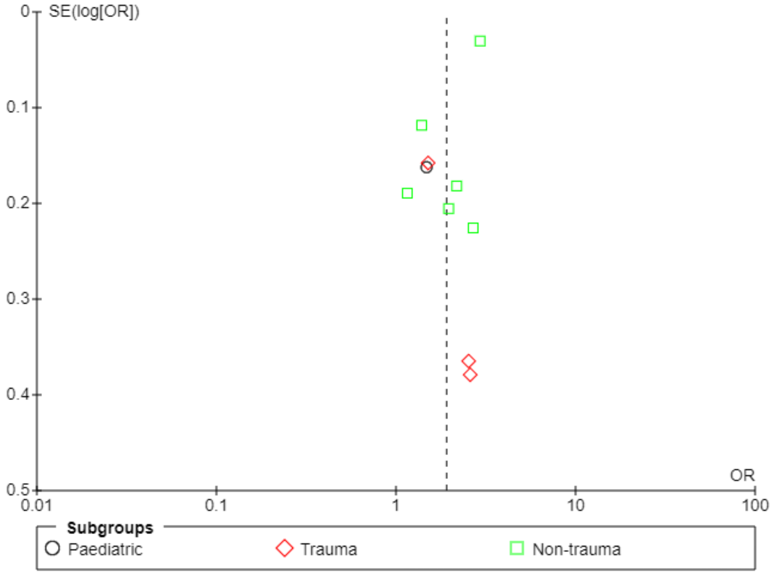

Survival to hospital discharge

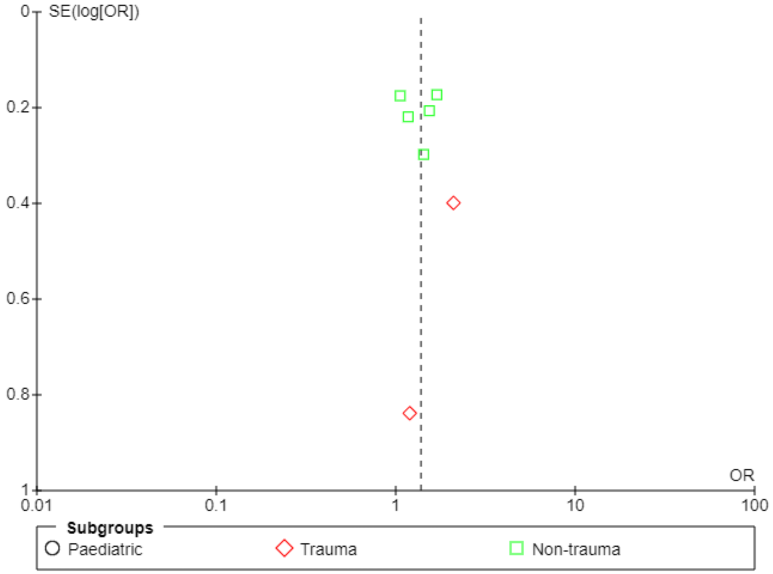

Survival at 30 days

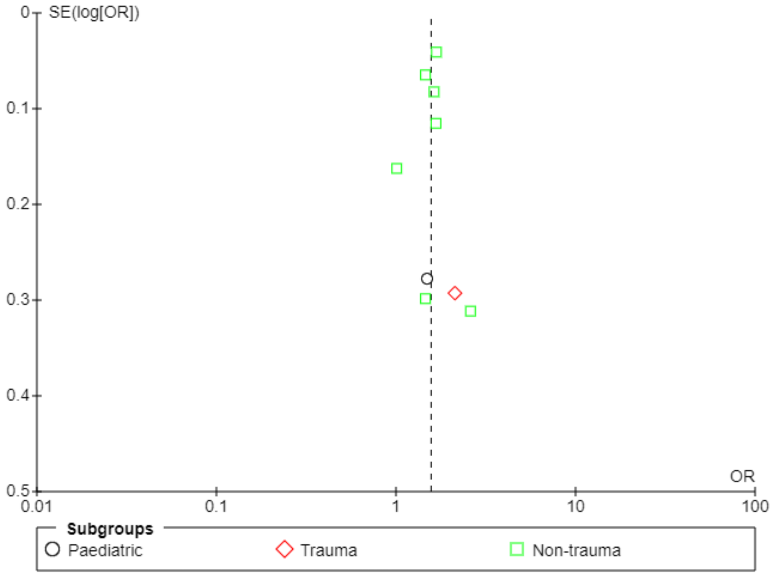

Favourable neurological outcome at 30 days

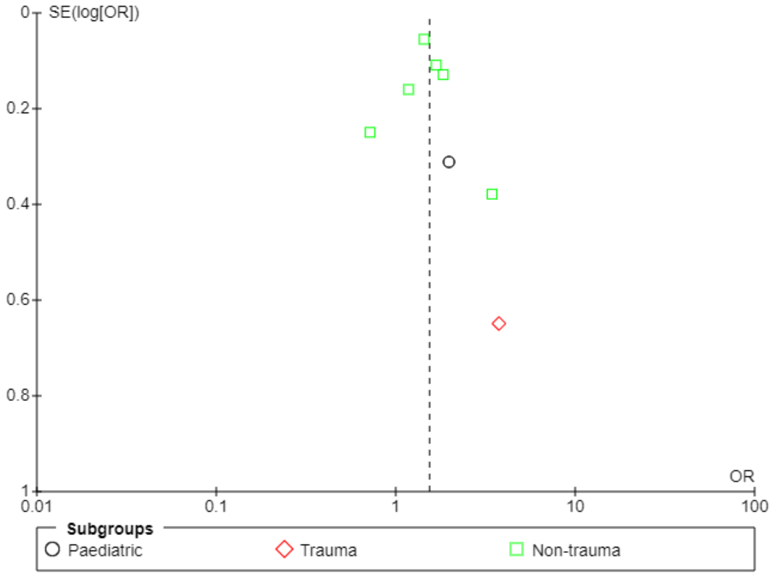

Supplement: Supplementary Data 5 [file mmc5.pdf]
