# Supplementary material for: Prehospital critical care beyond advanced life support for out-of-hospital cardiac arrest: A systematic review
Source: Resusc Plus. 2024 Dec 12;21:100803. doi: 10.1016/j.resplu.2024.100803 (PMC11728073; doi:10.1016/j.resplu.2024.100803)
Supplement: Supplementary Data 6 [file mmc6.docx]

**Supplementary Table 1** Patient and intervention characteristics

| **Author and year** | **Patient characteristics of CCT group** | **Patient characteristics of ALS group** | **Interventions in CCT group** | **Interventions in ALS group** |
| --- | --- | --- | --- | --- |
| Barnard 2019 [11] | Reported as total group only | Reported as total group only | Not reported | Not reported |
| Bjornsson 2021 [12] | N=200  Age: 67.6 (mean)  Male: 151 (75.5%)  Witnessed: 120 (60.0%)  Location: Not reported  Initial rhythm: Shockable 99 (49.5%)  Aetiology: All cardiac as per inclusion criteria  ByCPR: If witnessed, 74 (61.7%)  ByAED: Not reported  EMS response time: 6.3 min (mean) | N=271  Age: 69.7 (mean)  Male: 212 (78.2%)  Witnessed: 167 (61.6%)  Location: Not reported  Initial rhythm: Shockable 132 (48.7%)  Aetiology: All cardiac as per inclusion criteria  ByCPR: If witnessed, 102 (61.1%)  ByAED: Not reported  EMS response time: 7.2 min (mean) | Not reported | Not reported |
| Bujak 2022 [13] | N=351  Age: 66 (19)  Male: 244 (69.5%)  Witnessed: Not reported  Location: Home 258 (73.5%), Other 93 (26.5%)  Initial rhythm: Shockable 101 (28.8%)  Aetiology: Medical 309 (88.0%), other 42 (12.0%)  ByCPR: 172 (49.0%)  ByAED: Not reported  EMS response time: 9 min (6-12)** | N=461  Age: 67 (20)  Male: 327 (70.9%)  Witnessed: Not reported  Location: Home 347 (75.3%), Other 114 (24.7%)  Initial rhythm: Shockable 128 (27.8%)  Aetiology: Medical 423 (91.8%), other 38 (10.5%)  ByCPR: 253 (54.9%)  ByAED: Not reported  EMS response time: 8 min (6-10) | After PSM for baseline characteristics. Statistically significant differences:  Endotracheal intubation: 251 (75.4%)**  Amiodarone: 140 (43.1%)*  Atropine: 148 (45.5%)**  Time to termination of CPR: 32 min (21-42.5)** | After PSM for baseline characteristics  Endotracheal intubation: 195 (59.6%)  Amiodarone: 113 (35.5%)  Atropine: 104 (32.7%)  Time to termination of CPR: 37 min (28-49.5) |
| Dickinson 1997 [27] | N=9  Age: 69.6  Male: Not reported  Witnessed: Not reported  Location: Not reported  Initial rhythm: VF 5 (55.6%), PEA 3 (33.3%), Asystole 1 (11.1%)  Aetiology: Not reported  ByCPR: 2 (22.2%)  ByAED: Not reported  EMS response time: 6.6 min | N=40  Age: 67.5  Male: Not reported  Witnessed: Not reported  Location: Not reported  Initial rhythm: VF 22 (55.0%), PEA 15 (37.5%), Asystole 3 (7.5%)  Aetiology: Not reported  ByCPR: 8 (20.0%)  ByAED: Not reported  EMS response time: 5.1 min | Not reported | Not reported |
| Doan 2022 [28] | Reported as total group only | Reported as total group only | Reported as total group only | Reported as total group only |
| Fukuda 2018 [29] | N=828  Age: 58.1 (22.5) Mean (SD)  Male: 575 (69.4%)  Witnessed: 564 (68.1%)  Location: Not reported  Initial rhythm: VF 12 (1.4%), VT 1 (0.1%), PEA 306 (37.0%), asystole 444 (53.6%)*  Aetiology: All traumatic as per inclusion criteria  ByCPR: 179 (21.6%)  ByAED: Not reported  EMS response time: 9 min (7-12)** | N=1,591  Age: 59.2 (21.5) Mean (SD)  Male: 575 (69.4%)  Witnessed: 1129 (71.0%)  Location: Not reported  Initial rhythm: VF 39 (2.5%), VT 4 (0.3%), PEA 591 (37.1%), asystole 881 (55.4%)*  Aetiology: All traumatic as per inclusion criteria  ByCPR: 367 (23.1%)  ByAED: Not reported  EMS response time: 9 min (7-14) | Not reported | Not reported |
| Goto 2013 [30] | Reported as total group only | Reported as total group only | Not reported | Not reported |
| Goto 2019 [31] | N=19,551  Age: 68.8 (20.7)  Male: 12,244 (62.6%)  Witnessed: Bystander 9,036 (46.2%), EMS 2,272 (11.6%), Unwitnessed 8,243 (42.2%)  Location: Not reported  Initial rhythm: Shockable 2,217 (11.3%)  Aetiology: Presumed cardiac 10,249 (52.4%)  ByCPR: 9,300 (47.6%)  ByAED: 502 (2.6%)  EMS response time: 8.3 min (4.6) Mean (SD) | N=593,700  Age: 68.7 (20.1)  Male: 336,962 (56.8%)  Witnessed: Bystander 194,220 (32.7%), EMS 47,576 (8.0%), Unwitnessed 351,904 (59.3%)  Location: Not reported  Initial rhythm: Shockable 40,888 (6.7%)  Aetiology: Presumed cardiac 351,223 (59.2%)  ByCPR: 268,217 (45.2%)  ByAED: 6,152 (1.0%)  EMS response time: 7.8 min (3.7) Mean (SD) | Adrenaline 4,601 (23.5%) | Adrenaline 91,642 (15.4%) |
| Hatakeyama 2021 [15] | N=2,186  Age: 70.0 (15.3) Mean (SD)  Male: 1,438 (65.8%)  Witnessed: Bystander 1,286 (58.8%)  Location: Not reported  Initial rhythm: Shockable 530 (24.2%)  Aetiology: All medical as per inclusion criteria  ByCPR: 1,091 (49.9%)  ByAED: Not reported  EMS response time: 9.2 min (4.0) Mean (SD) | N=17,061  Age: 73.8 (14.9) Mean (SD)  Male: 10,513 (61.6%)  Witnessed: Bystander 7,595 (44.5%)  Location: Not reported  Initial rhythm: Shockable 1,883 (11.0%)  Aetiology: All medical as per inclusion criteria  ByCPR: 7,979 (46.8%)  ByAED: Not reported  EMS response time: 8.6 min (3.2) Mean (SD) | Not reported | Not reported |
| Nakajima 2023 [32] | N=316  Age: 59 (19)  Male: 265 (83.9%)  Witnessed: Bystander 245 (77.5%)  Location: Not reported  Initial rhythm: Shockable 236 (74.7%), PEA 51 (16.1%), Asystole 29 (9.2%)  Aetiology: Cardiac 279 (88.3%)  ByCPR: 166 (52.5%)  ByAED: Not reported  EMS response time: 9 min (3) | N=953  Age: 59 (20)  Male: 801 (84.1%)  Witnessed: Bystander 733 (76.9%)  Location: Not reported  Initial rhythm: Shockable 669 (70.2%), PEA 184 (19.3%), Asystole 100 (10.5%)  Aetiology: Cardiac 855 (89.7%)  ByCPR: 501 (52.6%)  ByAED: Not reported  EMS response time: 8 min (3) | Not reported | Not reported |
| Obara 2023 [33] | N=276  Age: 5.5 (14)  Male: 164 (59.4%)  Witnessed: 86 (35.0%)  Location: Not reported  Initial rhythm: Not reported  Aetiology: Cardiac 72 (26.1%)  ByCPR: 158 (64.2%)  ByAED: 3 (2.2%)  EMS response time: Not reported | N=911  Age: 2.0 (13)  Male: 559 (61.3%)  Witnessed: 250 (30.6%)  Location: Not reported  Initial rhythm: Not reported  Aetiology: Cardiac 292 (32.1%)  ByCPR: 460 (56.2%)  ByAED: 53 (5.8%)  EMS response time: Not reported | Not reported | Not reported |
| Olasveengen 2009 [34] | N=232  Age: 63 (18) Mean (SD)  Male: 160 (69.0%)  Witnessed: Bystander 145 (62.5%), EMS 9 (3.9%  Location: Home 107 (46.1%), Public 104 (44.8%), Other 20 (8.6%)  Initial rhythm: Shockable 90 (38.8%), Asystole 97 (41.8%), PEA 44 (19.0%)  Aetiology: Cardiac 168 (72.4%)  ByCPR: 141 (60.8%)  ByAED: Not reported  EMS response time: 9 min | N=741  Age: 65 (17) Mean (SD)  Male: 501 (67.6%)  Witnessed: Bystander 403 (54.4%), EMS 111 (15.0%)  Location: Home 444 (60.0%), Public 200 (27.0%), Other 96 (13.0%)  Initial rhythm: Shockable 220 (30.0%), Asystole 356 (48.0%), PEA 163 (22.0%)  Aetiology: Cardiac 497 (67.1%)  ByCPR: 387 (52.2%)  ByAED: Not reported  EMS response time: 9 min | Endotracheal intubation 205 (88.4%)**  Adrenaline 97 (41.8%)  Atropine 52 (22.4%)  Amiodarone 35 (15.1%)***  Defibrillation 109 (47.0%)**  Significantly improved hands-off ratio and pre-shock pause | Endotracheal intubation 582 (79%)  Adrenaline 321 (43.3)  Atropine 179 (24.2%)  Amiodarone 52 (7.0%)  Defibrillation 276 (37.2%) |
| Pemberton 2023 [16] | Reported as total group only | Reported as total group only | Not reported | Not reported |
| Sato 2019 [35] | N=135  Age: 66 (21)***  Male: 87 (64.4%)  Witnessed: All by-witnessed as per inclusion criteria  Location: Home 100 (74.1%), Work 7 (5.2%), Public 22 (16.3%), Other 6 (4.4%)  Initial rhythm: Shockable 51 (37.8%)***  Aetiology: Cardiac 90 (66.7%)  ByCPR: 81 (60.0%)*  ByAED: 3 (2.2%)  EMS response time: 8 min (3)* | N=757  Age: 78 (20)  Male: 510 (67.4%)  Witnessed: All by-witnessed as per inclusion criteria  Location: Home 558 (73.7%), Work 22 (2.9%), Public 136 (18.0%), Other 41 (5.4%)  Initial rhythm: Shockable 154 (20.3%)  Aetiology: Cardiac 461 (60.9%)  ByCPR: 372 (49.1%)  ByAED: 22 (2.9%)  EMS response time: 9 min (4) | Intravenous catheter insertion 110 (81.5%)**  Adrenaline 107 (79.3%)***  Time to adrenaline 20 min (9)* | Intravenous catheter insertion 527 (69.6%)  Adrenaline 436 (57.6%)  Time to adrenaline 22 min (9) |
| Von Vopelius-Feldt 2015 [36] | N=165  Age: 67 (23)***  Male: Not reported  Witnessed: 108 (65.5%)  Location: Home 101 (61.2%), Public 61 (37.0%)***  Initial rhythm: Shockable 68 (41.2%), PEA 23 (13.9%), Asystole 74 (44.8%)***  Aetiology: Not reported  ByCPR: 114 (69.1%)*  ByAED: Not reported  EMS response time: 6 min (5) | N=1,686  Age: 75 (20)  Male: Not reported  Witnessed: 1,040 (61.7%)  Location: Home 1,243 (73.7%), Public 237 (14.1%)  Initial rhythm: Shockable 451 (26.7%), PEA 390 (23.1%), Asystole 845 (50.1%)  Aetiology: Not reported  ByCPR: 1,015 (60.2%)  ByAED: Not reported  EMS response time: 6 min (5) | Not reported | Not reported |
| Von Vopelius-Feldt 2020 [10] | N=866  Age: 67 (18)***  Male: 611 (70.6%)***  Witnessed: Bystander 540 (62.4%), EMS 68 (7.9%), Unwitnessed 251 (29.0%)***  Location: Home 536 (61.9%), Public 225 (26.0%), Other 22 (2.5%)***  Initial rhythm: Shockable 305 (35.2%), PEA 130 (15.0%), Asystole 339 (39.2%)***  Aetiology: Cardiac 821 (94.8%)***  ByCPR: 620 (77.7%)***  ByAED: 54 (6.2%)***  EMS response time: 8.8 min (7.9)*** | N=7,149  Age: 74 (22)  Male: 4,549 (63.9%)  Witnessed: Bystander 3,506 (49.0%), EMS 1,225 (17.1%), Unwitnessed 2,390 (33.4%)  Location: Home 3,754 (52.5%), Public 758 (10.6%), Other 324 (4.5%)  Initial rhythm: Shockable 1,531 (21.4%), PEA 1,539 (21.5%), Asystole 3,542 (49.6%)  Aetiology: Cardiac 6,486 (90.7%)  ByCPR: 3,986 (67.3%)  ByAED: 233 (3.3%)  EMS response time: 7.2 min (6.2) | Interventions during arrest: Mechanical CPR 204 (39%), ROLE outside guidelines 124 (24%), Ultrasound 97 (19%), IV magnesium 31 (6%), IV bicarbonate 27 (5%), IV calcium chloride 15 (3%), thoracostomy 5 (1%).  Interventions after ROSC: RSI 94 (31%), IV inotropes or vasopressors 94 (31%), sedation and/or paralysis (not RSI) 64 (21%), Ultrasound 13 (4%), IV amiodarone 9 (3%), IV magnesium 7 (2%), IV bicarbonate 5 (2%), bypass nearest hospital for cardiac arrest centre 65 of 256 (25%) | Not reported |
| Yasunaga 2010 [37] | N=4,509  No bystander CPR group:  N= 1,597  Age: 65.9 (20.1)  Male: 1,058 (66.2%)  Witnessed: All by-witnessed as per inclusion criteria  Location: Not reported.  Initial rhythm: VF 245 (15.3%), PEA 573 (35.9%), Asystole 712 (44.6%), Other 67 (4.2%)  Aetiology: Cardiac 835 (52.3%)  Bystander CPR: 0 (0%)  Bystander AED: Not reported  EMS response time: 11.0 min (10.8)  Bystander CPR group:  N=1,916  Age: 69.2 (18.8)  Male: 1,180 (61.6%)  Witnessed: All by-witnessed as per inclusion criteria  Location: Not reported.  Initial rhythm: VF 424 (22.1%), PEA 627 (32.7%), Asystole 763 (39.8%), Other 102 (5.3%)  Aetiology: Cardiac 1,203 (62.8%)  ByCPR: 1,597 (100%)  ByAED: Not reported  EMS response time: 12.4 min (11.6) | N=113,690  No bystander CPR group:  N= 53,482  Age: 70.9 (17.4)  Male: 34,297 (64.1%)  Witnessed: All by-witnessed as per inclusion criteria  Location: Not reported.  Initial rhythm: VF 6,744 (12.6%), PEA 18,580 (34.7%), Asystole 27,129 (50.7%), Other 1,029 (1.9%)  Aetiology: Cardiac 28,823 (53.9%)  Bystander CPR: 0 (0%)  Bystander AED: Not reported  EMS response time: 10.9 min (9.9)  Bystander CPR group:  N=38,077  Age: 72.9 (18.1)  Male: 22,210 (58.3%)  Witnessed: All by-witnessed as per inclusion criteria  Location: Not reported.  Initial rhythm: VF 292 (16.5%), PEA 11,361 (29.8%), Asystole 18,724 (49.2%), Other 1,700 (4.5%)  Aetiology: Cardiac 21,967 (57.7%)  ByCPR: 38,077 (100%)  ByAED: Not reported  EMS response time: 11.7 min (9.7) | Not reported | Not reported |

**Supplementary Table 2** Study findings

| **Author and year** | **Study findings** |
| --- | --- |
| Barnard 2019 [11] | - Survival to hospital discharge significantly improved with CCT for non-trauma patients (adjOR 1.694, p=0.0023), but not for trauma patients (adjOR 1.197, p=0.83). - Survival to hospital admission significantly improved with CCT for non-trauma and trauma patients (adjOR 1.966, p<0.001; adjOR 2.591, p=0.012). |
| Bjornsson 2021 [12] | - No significant difference in survival to hospital discharge (25.0% vs 22.1%, p=0.456). - No significant differences in survival to hospital admission or ROSC (43.0% vs 39.5%, p=0.441; 53.5% vs 47.2%, p=0.180). - No outcome differences for initial rhythm subgroups. |
| Bujak 2022 [13] | - No significant difference in survival to hospital discharge with CCT (PSM OR 1.43, 0.80-2.57). - ROSC and survival to hospital admission significantly improved with CCT (PSM OR 1.49, 1.10-2.03; 2.18, 1.53-3.12). - CCT group had significantly increased advanced airway management and use of amiodarone (75.4% vs 59.6%; 43.1% vs 35.5%). CCT group had significantly decreased time to termination of CPR (32mins vs 37 mins). |
| Dickinson 1997 [27] | - Survival to hospital discharge significantly improved with CCT (44% vs 5%). - ROSC significantly improved with CCT (67% vs 30%). |
| Doan 2022 [28] | - No significant difference in survival to hospital discharge (adjOR 2.10, 0.96-4.57). - Survival to hospital admission significantly improved with CCT (adjOR 2.54, 1.25-5.18). |
| Fukuda 2018 [29] | - Survival with a favourable neurological outcome was significantly improved with CCT (adjOR 3.76, 1.14-14.51). - Survival at 30 days significantly improved with CCT (adjOR 2.21, 1.26-3.89). - Prehospital ROSC significantly improved with CCT (adjOR 1.51, 1.11-2.06). |
| Goto 2013 [30] | - Survival with a favourable neurological outcome was significantly improved with CCT (adjOR 1.68, 1.35-2.07). - Survival at 30 days significantly improved with CCT (adjOR 1.46, 1.28-1.65). |
| Goto 2019 [31] | - Survival with a favourable neurological outcome was significantly improved with CCT (adjOR 1.44, 1.29-1.60). - Survival at 30 days significantly improved with CCT (adjOR 1.68, 1.55-1.82). - Prehospital ROSC significantly improved with CCT (adjOR 2.94, 2.77-3.12). |
| Hatakeyama 2021 [15] | - Survival with a favourable neurological outcome was significantly improved with CCT (adjOR 1.84, 1.43-2.37). - Survival at 30 days significantly improved with CCT (adjOR 1.67, 1.33-2.09). - Similar results in non-shockable subgroup, but difference not statistically different in PSM adjusted shockable subgroup. |
| Nakajima 2023 [32] | - No significant difference in survival with a favourable neurological outcome (adjOR 0.72, 0.44-1.17). - No significant difference in survival at 30 days (adjOR 1.01, 0.73-1.38). |
| Obara 2023 [33] | - Survival with a favourable neurological outcome was significantly improved with CCT (adjOR 1.98, 1.08-3.66). - In-hospital ROSC significantly improved with CCT (adjOR 1.48, 1.08-2.04). - Survival at 30 days was not statistically significant (adjOR 1.49, 0.97-2.88). |
| Olasveengen 2009 [34] | - No significant difference in survival with a favourable neurological outcome (adjOR 1.35, 0.71-2.60). - No significant difference in survival to hospital discharge (13.4% vs 10.5%). - No differences in survival to hospital admission, or prehospital ROSC. - Similar results in subgroup of witnessed cardiac aetiology with shockable rhythm. |
| Pemberton 2023 [16] | - No significant difference in survival at 30 days (adjOR 1.46, 0.81-2.61). |
| Sato 2019 [35] | - Survival with a favourable neurological outcome was significantly improved with CCT (adjOR 3.44, 1.64-7.23). - Survival at 30 days significantly improved with CCT (adjOR 2.60, 1.41-4.78). - Prehospital ROSC significantly improved with CCT (adjOR 2.69, 1.73-4.19). |
| Von Vopelius-Feldt 2015 [36] | - No significant difference in survival to hospital discharge (adjOR 1.54, 0.89-2.67). |
| Von Vopelius-Feldt 2020 [10] | - No difference in survival to hospital discharge (PSM OR 1.06, 0.75-1.49). - Survival to hospital admission significantly improved with CCT (PSM OR 1.39, 1.10-1.75). - Critical care interventions in arrest were mechanical CPR (39%), termination outside of standard guidelines (24%), ultrasound (19%), and after ROSC were RSI (31%), inotropes/vasopressors (31%), bypass nearest hospital for CAC (25%), sedation and/or paralysis (not RSI) (21%). |
| Yasunaga 2010 [37] | - Survival at 30 days significantly improved with CCT (adjOR 1.63, 1.39-1.92). - No difference in favourable neurological outcome (adjOR 1.18, 0.86-1.61). |
